# Complete Supernumerary Nostril with Accessory Alar Fistula Reconstructed via Alar Wedge Excision Type 2: A Case Report and Literature Review

**DOI:** 10.1017/S0022215126104824

**Published:** 2026-07

**Authors:** Rizki Ekaputra Handoko, Iriana Maharani, Melody Audria Kurniadi

**Affiliations:** Otorhinolaryngology-Head and Neck Surgery, Universitas Brawijaya, Indonesia

**Keywords:** dacryocystorhinostomy, fistula, child, craniofacial abnormalities

## Abstract

**Background:**

Supernumerary nostril with an accessory nasal fistula is an exceptionally rare congenital anomaly with no standardised reconstructive protocol, especially with nasolacrimal involvement.

**Case report:**

A nine-year-old boy presented with a complete supernumerary nostril and synchronous accessory nasal fistula at the right alar base, persistent epiphora and prior endoscopic dacryocystorhinostomy. Imaging demonstrated a cystic tract communicating with the nasolacrimal system. Definitive management utilised a modified alar wedge excision type 2, incorporating both orifices into a single reconstructive unit. Three-month follow up demonstrated excellent aesthetic and functional outcomes with minimal scarring.

**Literature review:**

A review of 12 previously published cases (2001–2025) revealed heterogeneous, largely non-standardised techniques, often relying on simple excision that may not correct alar asymmetry.

**Conclusion:**

Complete tract eradication and restoration of alar symmetry were achieved. Alar wedge excision type 2 offers a reproducible technique for complex supernumerary nostril–accessory nasal fistula anomalies, particularly in Asian patients where tension-free closure minimises hypertrophic scarring.

## Introduction

Congenital nasal anomalies are exceptionally rare, occurring in less than 1 per cent of all congenital facial deformities.^1^ Among these, supernumerary nostril is one of the rarest malformations, characterised by the presence of an extra nasal orifice.^2^^,^^3^ Since its first description by Luschka in 1884, fewer than 50 cases have been documented worldwide, making it a diagnostic and surgical challenge for clinicians.^4^

Embryologically, these anomalies arise from disruptions in embryogenesis between the 3rd and 10th weeks of gestation, a period crucial for the formation of the nasal placode and the medial and lateral nasal processes.^5^^,^^6^ Primary hypotheses suggest that supernumerary nostril results from partial duplication of the nasal placode or a cleavage of the lateral nasal process during proliferation.^3^^,^^7^ Given that the lateral nasal process also contributes to the formation of the nasolacrimal duct, supernumerary nostril is frequently associated with other malformations, such as facial clefts or nasolacrimal duct obstruction.^8^ This association underscores the importance of a thorough pre-operative evaluation in all suspected cases.

Clinically, supernumerary nostril may present as a small, blind-ending pit or a complete canal communicating with the main nasal cavity.^3^^,^^4^ Because of its extreme rarity, it is often misidentified as a congenital nasal fistula, dermoid sinus or other naso-orbital anomaly.^8^^,^^9^ While surgical intervention is universally recommended to restore aesthetics and prevent complications such as recurrent infection, no universally accepted classification or standardised surgical taxonomy currently exists.^2^^,^^4^ The emergence of refined surgical techniques, such as standardised alar base surgery, provides a framework for achieving optimal symmetry.^10^^,^^11^ This article reports a unique synchronous presentation of a complete supernumerary nostril and an accessory alar fistula in a paediatric patient, managed via a standardised alar wedge excision type 2 approach, followed by a brief review of the literature.

## Case presentation

**Figure 1.**
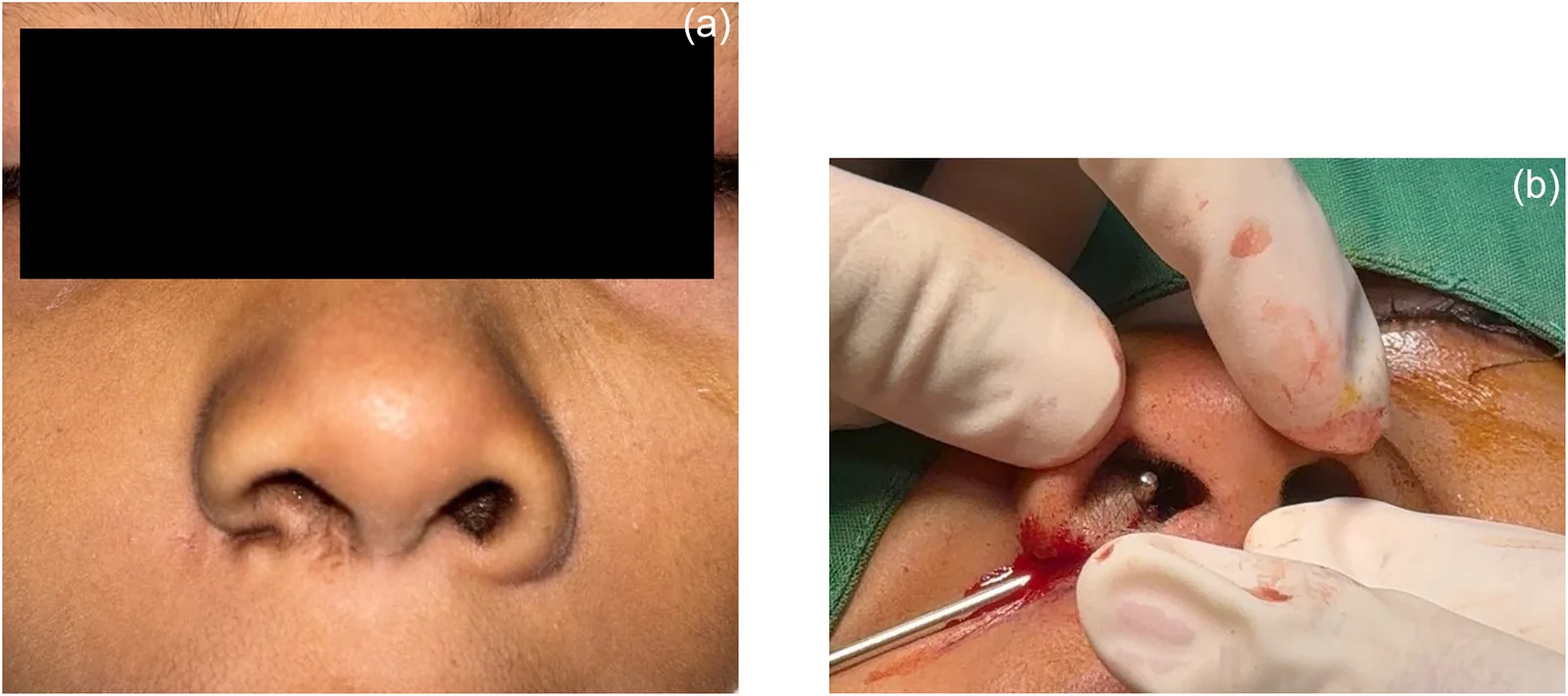
Pre-operative clinical presentation of the nine-year-old male patient. (a) Frontal view illustrating the complete supernumerary nostril at the right alar base. (b) Close-up view showing the synchronous accessory nasal fistula situated laterally to the primary orifice and the associated alar base asymmetry. Figure 1 long description.

A nine-year-old male was referred to our tertiary otorhinolaryngology department with a congenital opening on the right alar base (Figure 1). The patient’s parents reported that a small orifice had been present since birth, intermittently discharging clear or yellowish mucoid material, particularly during episodes of upper respiratory tract infections. Notably, the mother observed that fluid could be expressed from the right eye on applying local pressure over the nasal region. The patient had a significant history of persistent epiphora and had been diagnosed with total nasolacrimal duct obstruction at a secondary hospital. Consequently, an endoscopic dacryocystorhinostomy (DCR) had been performed in August 2025 to address the lacrimal symptoms.

Physical examination revealed two distinct abnormal orifices on the right alar region. The primary orifice, located medially near the alar base, was identified as a complete supernumerary nostril, while a second, smaller orifice situated more laterally was consistent with an accessory nasal fistula. Significant asymmetry of the alar base was noted compared with the contralateral side. Internal nasal examination and systemic evaluations were otherwise unremarkable.

Pre-operative imaging provided crucial anatomical details. A computed tomography scan of the paranasal sinuses demonstrated a cystic lesion in the right nasal region that appeared to communicate with the nasolacrimal duct, without evidence of intracranial extension. Right-sided maxillary and ethmoid sinusitis were also noted. Dacriography confirmed a total obstruction at the proximal level of the right nasolacrimal duct. Based on the clinical and radiological findings, a diagnosis of a complete supernumerary nostril associated with a synchronous accessory nasal fistula was established.

**Figure 2.**
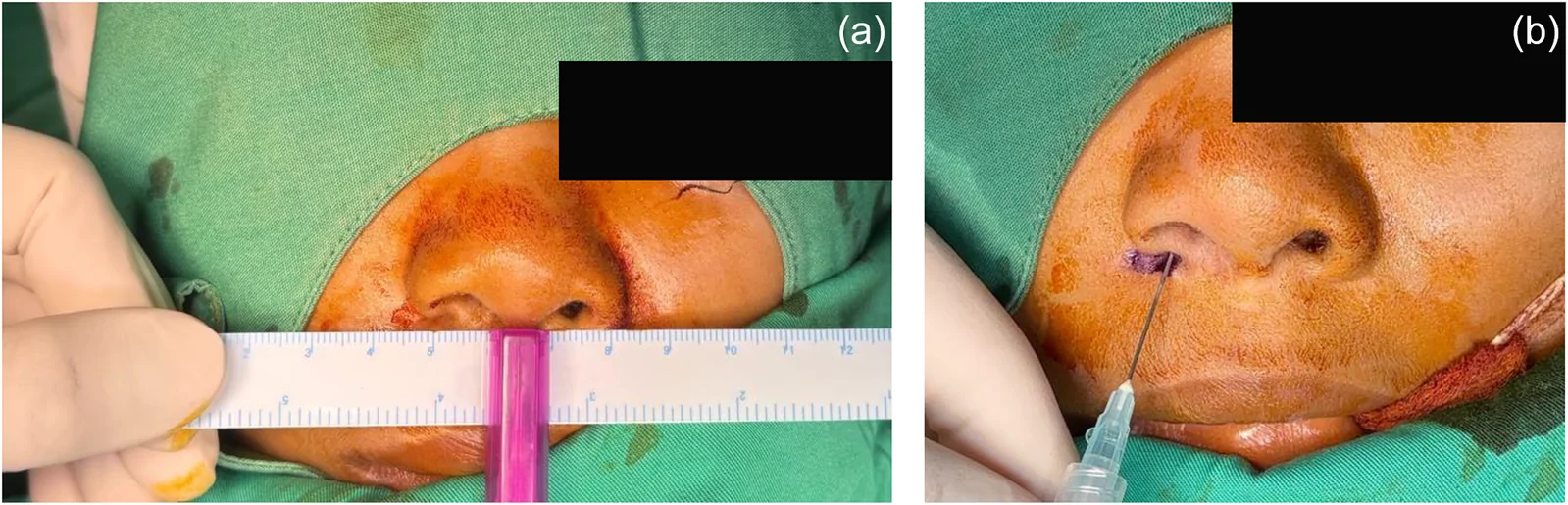
Intra-operative surgical markings. The adapted alar wedge excision type 2 design is outlined to encompass both the supernumerary nostril and the accessory fistula within a single reconstructive unit. Figure 2 long description.

The patient underwent definitive surgical management in October 2025. Under general anaesthesia, a total fistulectomy was performed in conjunction with a reconstruction using an alar wedge excision type 2. A wedge-shaped incision was meticulously designed to encompass both the supernumerary nostril and accessory nasal fistula orifices (Figure 2).

**Figure 3.**
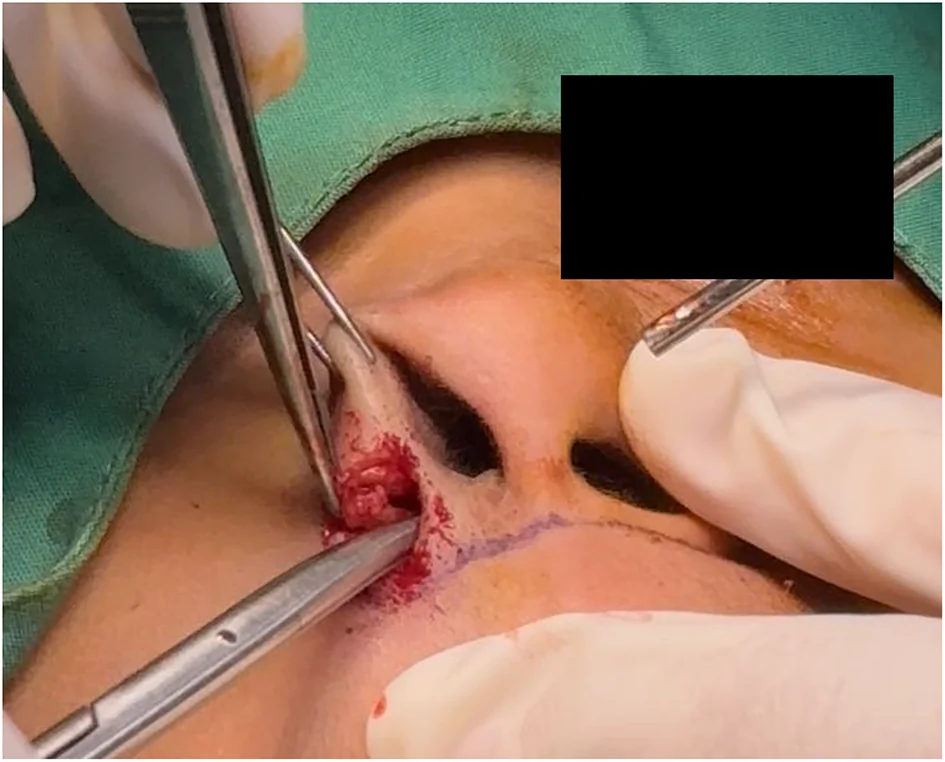
Intra-operative dissection. Detailed tracking and total excision of the epithelialised tract extending towards the junction of the inferior turbinate and the limen nasi. Figure 3 long description.

**Figure 4.**
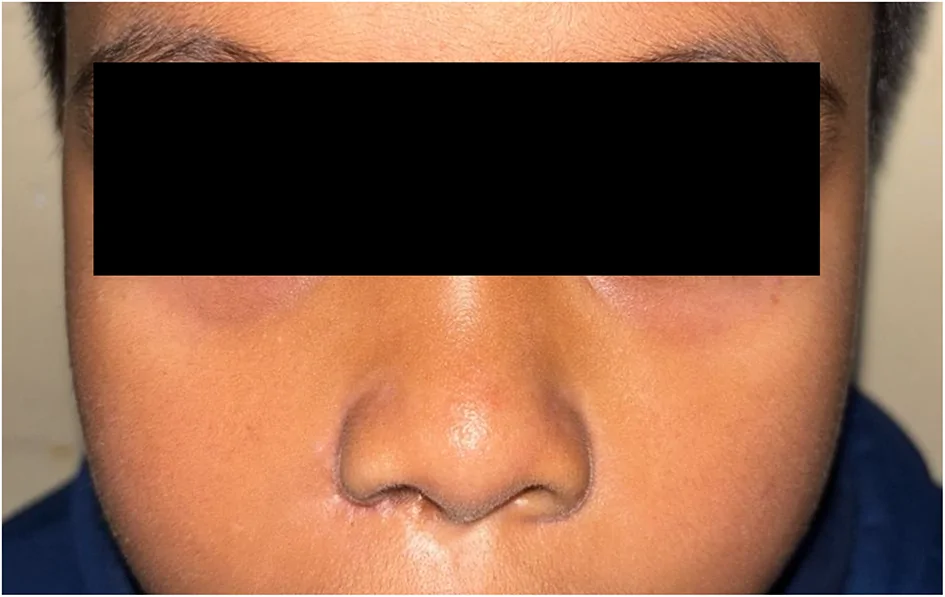
Post-operative outcome at three months. The images demonstrate stable restoration of alar symmetry, complete resolution of the cystic lesion symptoms and excellent scar camouflage within the natural alar-facial grooves.

The epithelialised tract was tracked and dissected to its termination at the junction of the inferior turbinate and the limen nasi, where it was completely excised (Figure 3). Primary closure was performed in layers using 5.0 and 6.0 sutures to restore the aesthetic contour and achieve alar symmetry. Post-operative evaluation at 14 days, 21 days and 3 months (Figure 4) demonstrated excellent wound healing with no immediate complications (haemorrhage, haematoma or infection), significant restoration of alar base symmetry, satisfying aesthetic contours with zero functional complaints (discharge, obstruction or epiphora) and high parental satisfaction regarding the stable cosmetic outcome.

## Literature review

A review of 12 published cases (2001–2025) demonstrates the lack of consensus regarding the management of supernumerary nostril. Although rare, the condition is often associated with alar base asymmetry and, in some cases, nasolacrimal duct anomalies Table 1. Simple fistulectomy or elliptical excision has been the most commonly reported treatment. However, these reductive approaches may result in alar notching and persistent asymmetry, particularly in complete duplication variants.^12^^,^^13^ The diverse nomenclature used across the literature has further hindered the development of a standardised reconstructive algorithm.^14^^–^^16^ Achieving long-term anthropometric symmetry remains the most significant challenge in paediatric populations.^17^^,^^18^

**Table 1.** Summary of clinical characteristics and surgical interventions between the present case and curated studies (*n* = 12) Table 1 long description.

No	Reference	Age, sex	SN type	Surgical technique	Key outcome
–	Present case	9, M	Complete SN	Alar wedge excision type 2	Excellent symmetry
1	Uppal *et al*. (2011)^3^	8, M	Isolated SN	Wedge excision	Alar flare control
2	Hallak *et al*. (2001)^4^	2, M	Complete SN	Simple excision	No recurrence
3	Srivastava & Kumar (2023)^8^	5, F	Isolated SN	Total fistulectomy	No recurrence
4	Li *et al*. (2022)^9^	12, M	Concealed SN	Excision and local flap	Contour restoration
5	Choi *et al*. (2016)^11^	15, M	Complete SN	Elliptical excision	Satisfactory result
6	Widarda *et al*. (2024)^12^	4, M	Complete SN	Alar reconstruction	Aesthetic success
7	Alqabbani *et al*. (2020)^13^	22, M	Adult SN	Surgical excision	Functional clearance
8	Al Hafiz *et al*. (2025)^14^	10, M	Functional SN	Open rhinoplasty	Structural support
9	Tuncbilek *et al*. (2005)^15^	3, F	Isolated SN	Z-plasty reconstruction	Improved symmetry
10	van Wingerden (2008)^16^	2, F	Incomplete SN	Cartilaginous repair	Structural integrity
11	Kim *et al*. (2016)^17^	1, F	Isolated SN	Elliptical excision	Minimal scarring
12	Reddy *et al*. (2016)^18^	2, M	Complete SN	Simple excision	Satisfactory result

SN = supernumerary nostril; M = male; F = female

## Discussion

The synchronous presentation of a complete supernumerary nostril and an accessory nasal fistula in a nine-year-old patient represents an exceptionally rare clinical event that transcends simple morphological duplication. Since Luschka’s initial description in 1884, the rarity of this condition has limited the medical community’s ability to establish a robust evidence-based surgical protocol.^3^^,^^4^ Morphogenetically, this anomaly is best understood within the framework of a segmental duplication or an aberrant cleavage of the lateral nasal process during the critical period of facial ontogeny between the fifth and eighth weeks of gestation.^5^^,^^6^

The clinical triad observed in our patient, comprising a supernumerary nostril, an accessory fistula and nasolacrimal duct obstruction, underscores a profound developmental disruption along the nasolacrimal groove. This confirms that the defect is not merely a superficial cutaneous anomaly but a complex structural malformation originating from the failure of proper mesenchymal fusion between the lateral nasal and maxillary processes.^7^^,^^9^ The necessity of a prior endoscopic DCR in this case further highlights the profound involvement of the nasolacrimal system. This multi-staged approach addressing the functional lacrimal obstruction before definitive alar reconstruction is crucial for managing the complex clinical triad often associated with failures in mesenchymal fusion along the nasolacrimal groove.^8^^,^^9^

Historical literature predominantly advocates for simple elliptical excision or fistulectomy. However, these reductive approaches often neglect the intricate three-dimensional architecture of the alar base.^8^^,^^11^ Our analysis of the high-quality evidence, including recent reports by Widarda *et al*. and Alqabbani *et al*., suggests that traditional excision techniques are frequently associated with post-operative alar notching and persistent asymmetry, particularly in complete duplication variants.^12^^,^^13^ Furthermore, the diverse nomenclature used across the literature ranging from ‘accessory nostrils’ to ‘functional supernumerary nostril’ has historically hindered the development of a standardised reconstructive algorithm.^14^^–^^16^ The findings from these curated studies highlight that achieving long-term anthropometric symmetry remains the most significant challenge, especially in paediatric populations.^17^^,^^18^

In response to these challenges, the implementation of the modified alar wedge excision type 2 represents a significant paradigm shift from simple destructive surgery to a vector-based anatomical reconstruction. Originally standardised by Rohrich *et al*. for aesthetic rhinoplasty, this technique was strategically adapted in our case to address the structural redundancy of the alar base.^10^ By meticulously designing the wedge to encompass both the supernumerary orifice and the accessory fistula, we facilitated a comprehensive fistulectomy while simultaneously managing the excess alar flare. From a biomechanical perspective, the type 2 wedge excision allows for the redistribution of surgical tension along the natural anatomical boundaries of the alar-facial groove. This is particularly advantageous in patients of Asian descent, where the thicker dermal layer and increased fibroblastic activity necessitate a tension-free closure to mitigate the risk of hypertrophic scarring.^19^^,^^20^

The successful restoration of the alar-facial subunits in our patient, sustained at a three-month follow up, suggests that the integration of standardised rhinoplasty principles into congenital reconstructive surgery is essential. The absence of hypertrophic scarring or alar notching at this stage provides a compelling argument for the stability of this approach. Although the current evidence is constrained by the inherent limitations of case-based reviews, the clinical success of this approach provides a compelling argument for moving beyond simple fistulectomy. The synthesis of our findings with the curated literature suggests that a tailored, subunit-based reconstruction not only ensures the complete eradication of the epithelialised tract but also addresses the profound psychological and aesthetic implications of congenital nasal duplication. Future longitudinal studies are warranted to evaluate the stability of these reconstructive results as the paediatric facial skeleton matures.^21^

- Supernumerary nostril is an exceedingly rare congenital malformation, with fewer than 50 cases documented globally since 1884
- Surgical management typically involves simple fistulectomy or tract excision, but standard protocols for achieving alar symmetry remain limited
- Associated anomalies, particularly those involving the nasolacrimal system, are recognised but often under-reported in individual case studies
- This study reports a unique synchronous presentation of complete supernumerary nostril and an accessory nasal fistula in a nine-year-old male, associated with total nasolacrimal duct obstruction, and demonstrates that a single stage alar wedge excision type 2 can simultaneously eradicate both epithelialised tracts and correct alar base asymmetry in a paediatric patient
- Twelve published cases are reviewed, showing that reconstruction techniques achieve superior aesthetic outcomes compared with simple excision
- The clinical utility of the alar wedge excision type 2 approach is demonstrated as a standardised reconstructive approach to optimise both functional tract removal and aesthetic alar symmetry

## Conclusion

The synchronous presentation of a complete supernumerary nostril associated with an accessory nasal fistula is an exceptionally rare clinical entity that necessitates a sophisticated reconstructive strategy beyond simple destructive excision. The adaptation of the alar wedge excision type 2 provides a robust and reproducible framework for achieving total excision of the epithelialised tract while simultaneously restoring alar symmetry. This subunit-based approach is particularly effective in managing the thicker dermal characteristics of the Asian nose, allowing for tension-free closure within natural anatomical creases and minimising the risk of hypertrophic scarring. For the paediatric population, transitioning towards such standardised reconstructive algorithms is essential to ensure long-term aesthetic stability and psychosocial well-being as the facial skeleton matures.

## Figure 1 Long description

A composite of two clinical photographs. A) A close-up frontal view of the lower nose and upper lip. Two nostril openings are shown. The label “(a)” is printed near the top right. B) A close-up view of the side of the nose in a surgical field. Two gloved hands hold and retract tissue at a nostril opening while a metal instrument is positioned at the opening. Blood is present at the opening and on the surrounding skin. A surgical drape is present around the area. The label “(b)” is printed near the top right.

Navigate back to Figure 1.

## Figure 2 Long description

The image contains two clinical photographs, labeled (a) and (b), showing surgical procedures. Image (a) shows a ruler being held horizontally across the nose. The ruler is white with black markings and a pink vertical support. Image (b) shows a needle being inserted into the nose near the nostril. The needle is attached to a clear plastic syringe.

Navigate back to Figure 2.

## Figure 3 Long description

A close-up shot shows a surgical setting where gloved hands are manipulating surgical instruments. The instruments are positioned around an area with some visible markings.

Navigate back to Figure 3.

## Table 1 Long description

The table compares 13 reported cases of supernumerary nostril, listing each reference, patient age and sex, the described type, the surgical technique used, and the main reported outcome. Ages range from 1 to 22 years, with most patients being male. Reported types include complete, isolated, concealed, incomplete, functional, and adult presentations. Techniques are mainly excision-based approaches such as wedge, simple, or elliptical excision, with several reconstructive methods including local flap, Z-plasty, cartilaginous repair, alar reconstruction, and open rhinoplasty. Outcomes are generally positive, commonly described as no recurrence, satisfactory result, improved or excellent symmetry, contour restoration, functional clearance, or structural support. The present case involves a 9-year-old male with a complete type treated with an alar wedge excision type 2, reported as excellent symmetry. Interpretation is limited because outcomes are qualitative and terminology varies across reports, so direct comparisons of effectiveness are not standardized.

Navigate back to Table 1.
